# Associations between physical activity, sleep patterns and diet quality and menstrual health symptoms in midlife: evidence from the 1970 British Cohort Study

**DOI:** 10.1186/s12905-026-04533-9

**Published:** 2026-05-26

**Authors:** Kamilla Abdullayev, John Joseph Mitchell, Joanna M Blodgett

**Affiliations:** 1 Institute of Sport Exercise & Health, Division of Surgery and Interventional Sciences, Faculty of Medical Sciences, University College London, London, UK; 2 Department of Primary Care and Population Health, Upper Third Floor UCL Medical School (Royal Free Campus), Rowland Hill Street, London, NW3 2PF UK; 3 Biomedical Research Centre, University College London Hospitals NIHR, London, UK

**Keywords:** Menstrual Health, Lifestyle Behaviours, Sleep, Physical Activity, Diet, Midlife, Perimenopause

## Abstract

**Background:**

Menstrual health symptoms, including painful periods, heavy bleeding, and premenstrual syndrome (PMS), are prevalent in midlife women and may be influenced by lifestyle factors. Identifying modifiable health behaviours associated with these symptoms could inform non-pharmacological interventions. This study examined the associations between diet quality, physical activity, and sleep patterns with menstrual health symptoms in midlife women using data from the 1970 British Cohort Study.

**Methods:**

Participants (*N* = 2,109) were drawn from the age 46 sweep of the 1970 British Cohort Study, a birth cohort of individuals born within a single week. Dietary data was collected using the 24-hour Oxford WebQ questionnaire and a Mediterranean diet score was derived as an indicator of diet quality. Physical activity (total time and moderate-vigorous physical activity) and sleep patterns (duration, efficiency, regularity) were measured via thigh-worn accelerometers for a 7-day wear period. Quartiles were derived for each continuous exposure, with the healthiest quartile as the reference group. Binary menstrual health outcomes (heavy periods, painful periods, PMS) were self-reported. Binomial logistic regression models estimated associations between individual lifestyle factors and each menstrual symptom, adjusting for irregular menstrual bleeding, contraception use, body mass index (BMI), education, endometriosis diagnosis, smoking status and cohabiting status.

**Results:**

Approximately half of participants reported experiencing painful periods (44.1%), heavy periods (51.8%) or PMS (49.5%). Participants in the lowest quartile of diet quality had reduced odds of experiencing PMS symptoms (adjusted OR: 0.75, 95% confidence interval (CI): 0.57, 0.98)). There was no significant association between total physical activity and any of the menstrual symptoms in adjusted models, however, those in the lowest quartile of moderate-vigorous physical activity had 1.35 (1.01, 1.80) times higher odds of having painful periods and 1.33 (0.99, 1.79) times higher odds of having heavy periods in adjusted models. There were no associations with PMS and any physical activity variable. Those in the lowest sleep regularity quartile had 1.43 (1.08,1.91) higher odds of experiencing painful periods, 1.34 (1.00,1.79) higher odds of heavy periods and 1.33 (1.01,1.76) higher odds of PMS in adjusted models. Lowest levels of sleep efficiency showed 1.41 (1.07,1.84) higher odds of painful periods in unadjusted models. Sleep duration was not associated with any menstrual health outcomes.

**Conclusions:**

Findings suggest that specific lifestyle behaviours, such as moderate-vigorous physical activity and sleep regularity, are associated with lower risk of painful periods, with further evidence indicating sleep regularity is also associated with heavy periods and PMS. Future research should explore potential causal relationships and intervention strategies to improve menstrual health through lifestyle modifications.

**Supplementary Information:**

The online version contains supplementary material available at 10.1186/s12905-026-04533-9.

## Introduction

Menstrual symptoms can significantly impair daily functioning across social, workplace, and educational settings [1–3] and are associated with poorer quality of life and wellbeing [4, 5]. Research has historically overlooked midlife menstrual experiences in favour of early reproductive years and fertility [6, 7]. However, this transitional phase leading to menopause is characterised by hormonal fluctuations that may impact physiological processes, leading to heavier and more irregular bleeding, increased pain, and worsened premenstrual syndrome (PMS) [8, 9]. These exacerbations not only disrupt daily activities and diminish quality of life [10] but can also signal broader health risks, including increased susceptibility to various chronic diseases [11, 12]. Given the increased symptom burden during perimenopause [13, 14], lifestyle-based interventions offer a promising alternative to pharmacological treatments, which may not be suitable or accessible for all individuals [15, 16]. While strong evidence links alcohol consumption and smoking to menstrual disturbances via inflammatory and hormonal pathways [17–19], research on the importance of other lifestyle behaviours such as diet quality, physical activity and sleep remains mixed.

Prior studies have linked the Mediterranean diet to a reduced risk of painful and heavy periods, as well as PMS symptoms [20–23]. Moreover, emerging research suggests that dietary patterns rich in whole grains, healthy fats, and phytoestrogens, such as those found in the Mediterranean diet, may alleviate perimenopausal symptoms [24]. However, generalisability of existing studies is limited by the reliance on poor quality dietary measures, cross-sectional designs and small sample sizes.

Physical activity has also been linked to menstrual symptom experiences, with a recent systematic review of 82 observational studies revealing an increased risk of menstrual pain and PMS severity in those engaging in lower levels of physical activity [25]. Several systematic reviews and meta-analyses have consistently demonstrated that exercise can reduce prevalence and severity of PMS and menstrual symptoms [26–28]. The benefits of PA also appear to extend beyond menstrual symptoms, as increased physical activity levels have been associated with reduced psychosocial and physical menstrual and perimenopausal symptoms across both observational studies and community-based interventions [29, 30]. Despite these promising findings, research in this area is limited by the widespread reliance on self-reported physical activity measures, which are subject to desirability bias and failure to provide information on activity intensity, emphasising the need for device-based measures [25].

Finally, findings from a recent systematic review have linked poorer sleep quality, increased daytime sleepiness, and reduced sleep efficiency to menstrual symptoms, particularly PMS and painful periods, though findings for sleep duration are inconsistent [31]. Given the high prevalence of sleep disruptions during perimenopause [32], it is important to understand how different sleep characteristics may contribute to menstrual symptom experiences to help improve sleep guidance and interventions. However, while subjective assessments provide insight into perceived sleep quality, studies incorporating objective measures, such as actigraphy and polysomnography, have reported discrepancies in findings [33], emphasising the need for device-measured sleep data to capture more precise sleep characteristics.

Despite growing acknowledgement of lifestyle influences on menstrual health, research in midlife women remains limited and often of poor quality, despite this critical reproductive life stage [8, 9]. Identifying modifiable risk factors can help provide effective therapeutic targets for behavioural interventions [34]. This study aimed to examine associations between diet quality, device-measured PA, and sleep patterns with three common menstrual symptoms in midlife women: painful periods, heavy bleeding, and PMS.

## Methods

### Study sample

The 1970 British Cohort Study is a population-based study of over 17,000 individuals born in the same week in April 1970, followed at 10 + time points [34]. This study utilises data from the age 46 sweep, which collected data via self-completed questionnaires, computer-assisted interviews, a physical assessment by a trained nurse, and a 7-day accelerometer protocol [34]. Ethical approval was granted by the Southeast Coast Brighton and Sussex Ethics Committee (15/LO/1446) and all participants provided informed written consent.

**Fig. 1:**
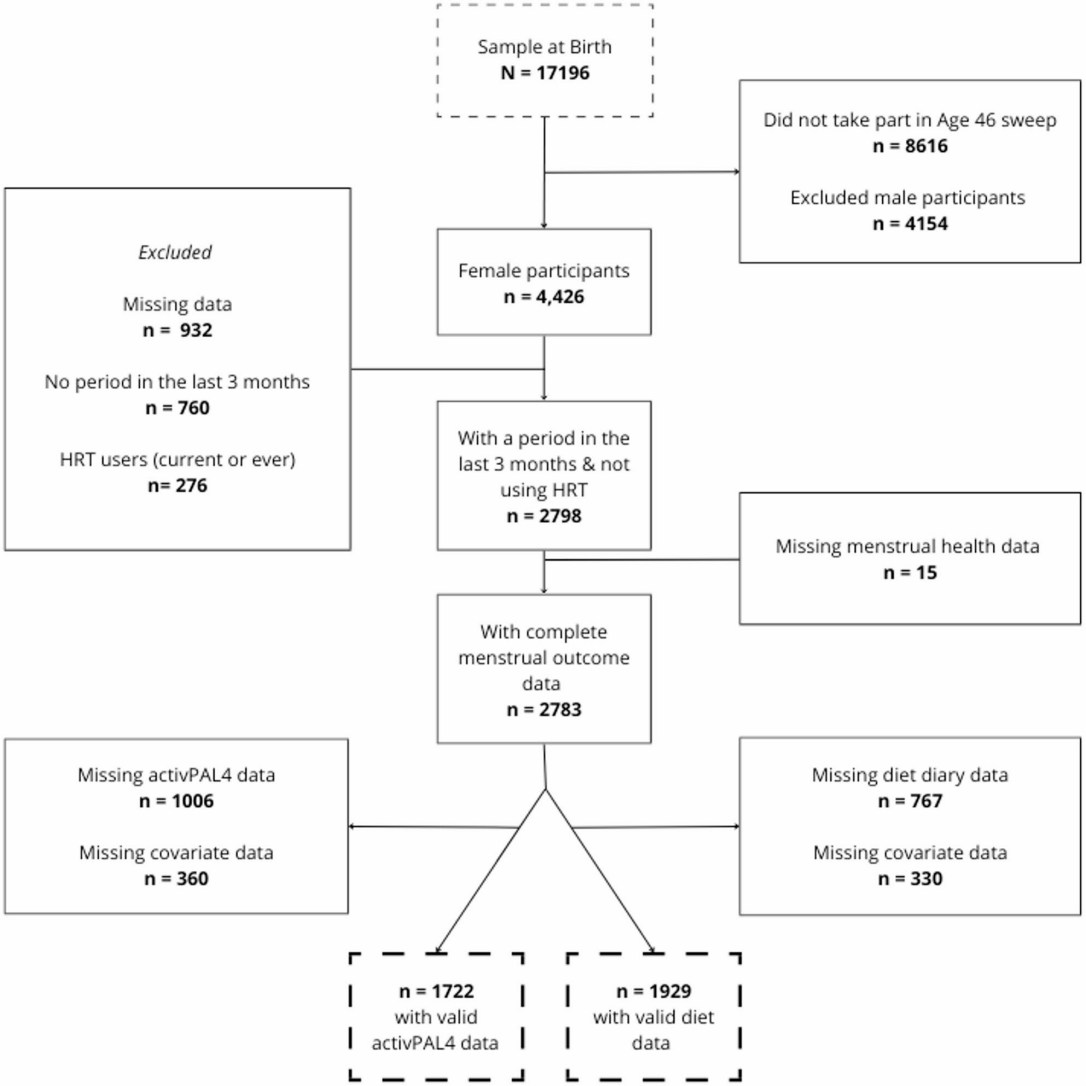
Derivation of the final analytic samples (excluded participant groups are not mutually exclusive)

Of 17,196 participants included in BCS70 at birth, 8,581 took part in the age 46 cohort and 6,492 participants consented to wear the thigh-worn accelerometer, with 5,569 returning monitors with at least one day of usable data. Participants were excluded if they were male, reported not having a period in the last 3 months, were currently on hormone replacement therapy (HRT) or had ever been on HRT, had missing menstrual health symptom data, or any missing covariate data. To maximise sample size, two separate analytic samples were used based on availability of diet diary data and accelerometery data (measuring sleep patterns and physical activity, more detail below). Figure 1 presents the full flowchart of the sample derivation process.

### Exposures

Free-living physical activity and sleep patterns were recorded by a thigh-mounted activPAL4 micro triaxial accelerometer (PAL Technologies Ltd., Glasgow, United Kingdom). The waterproof device was fitted on the midline anterior aspect of the upper thigh by a trained nurse and participants were instructed to wear it for seven days without removal. Participants were instructed not to reattach the device if it fell off before the period ended [35]. Raw accelerometer data was processed using the ActiPASS software, which classifies accelerometer signals in two-second windows with a 50% overlap using a decision tree [36]. The algorithm included non-wear detection, sleep classification, posture identification, and activity intensity, where intensity thresholds were based on established cadence criteria [37–39]. Wear time was distinguished from non-wear using standardised protocols [40, 41]. Sleep periods were then identified using a separate decision tree algorithm, which had been validated against polysomnography with accuracy greater than 0.86 [42]. Following this, an activity classifier was used to detect specific activities and postures based on signal standard deviation and tilt angle.

#### Physical activity

In addition to total daily physical activity time, daily moderate-vigorous physical activity time was categorised using predefined thresholds for stepping intensity (≥ 100 steps/min) [42–47]. Both measures are reported in minutes/day. Participants with at least one wear day, more than one detected walking period, and > 0 min of sleep were included in the final accelerometer data sample [48].

#### Sleep

Three sleep measures – sleep duration, efficiency, and regularity – were derived using an accelerometer-based sleep estimation algorithm [42]. Sleep duration (total hours) was derived using a validated algorithm that identified thresholds for wakefulness and sleep [49]. Sleep efficiency, expressed as a percentage, represents the proportion of time asleep relative to time in bed [50]. Sleep regularity was measured using the Sleep Regularity Index (SRI), representing the probability of being in the same state (sleep/wake) at the same time on consecutive days, with higher percentage values indicating greater regularity [51].

#### Diet quality

Dietary intake was assessed using the previously validated Oxford WebQ diet diary [52]. Participants reported all food and drink consumption for the previous day, completing the diary twice—once on a weekday and once on a weekend day. A pyramid-based Mediterranean diet quality score (PyrMDS) was calculated, incorporating 15 food components assigned a continuous score from 0 to 1 based on recommended daily consumption [53] (Table S1 for the breakdown of food groups). The outcome score considers the consumption of inter-related nutrients and foods, and both the traditional Mediterranean diet and contemporary food environment [54]. The total PyrMDS ranged from 0 to 15, with higher scores indicating better adherence to the Mediterranean diet and thus higher diet quality [55].

### Outcomes

Participants self-reported whether they experienced three menstrual symptoms experienced in the past four years: heavy periods, painful periods, and premenstrual symptoms (PMS). Response options for each were binary (yes/no).

### Covariates

Covariates were selected based on known associations with physical activity, diet quality, sleep, and menstrual health, including irregular menstrual bleeding, contraception use, body mass index (BMI), highest educational qualification, endometriosis diagnosis, smoking status, and cohabiting status [56–63].

Irregular bleeding was assessed via a yes/no response. Contraception use was determined by whether participants had taken oral contraceptives or used injections/implants in the past year (yes/no). BMI was calculated from nurse-measured height and weight (kg/m²). Educational attainment was collapsed from eight categories into five categories: No Academic Qualifications, GCSEs or equivalent (typically attained at 16 years), A Levels or equivalent (typically attained at 18yrs), Diploma, and Degree or above. Endometriosis diagnosis was self-reported (yes/no). Smoking status included four categories: Never smokers, Ex-smokers, Occasional smokers, Everyday smokers. Cohabiting status was self-reported and collapsed into four categories: living with partner, living with civil partner, living with spouse, not living with partner/no partner.

### Statistical analyses

Complete cases only were used, hence participants with missing data on menstrual health symptoms or any covariates were excluded from the final analysis. Sample characteristics were summarised using means and standard deviations for continuous variables and frequencies and percentages for categorical variables. Binomial logistic regression assessed associations between each individual lifestyle factor (diet quality, physical activity, and sleep) and each menstrual symptom outcome. To address non-linearity, continuous exposure variables were categorized into quartiles, using the healthiest quartile as the reference. Both unadjusted and adjusted models (adjusted for contraception use, having irregular periods, an endometriosis diagnosis, BMI, smoking status, highest educational attainment, and cohabiting status) are reported. Independent t-tests and chi-square tests were used to compare the included and excluded participants on their descriptive characteristics. Further sensitivity analyses were conducted with number of children as an additional covariate to capture potential care burden associated with parity in addition to household support captured via cohabiting status; these were not included in main models due to concerns of overadjustment( Table S4). All analyses were conducted using R (version 4.4.2) and statistical significance was set at α < 0.05.

## Results

### Sample characteristics

Approximately half of the 2,109 included participants reported experiencing each of painful periods (44.1%, *n* = 931), heavy periods (51.8%, *n* = 1093) or PMS (49.5%, *n* = 1044). Symptom co-occurrence was common, with a quarter reporting all three symptoms (25.4%, *n* = 535) and only 28.4% reporting no symptoms at all (*n* = 599). Sample characteristics are presented in Table 1. Overall, participants had high educational attainment (48.7% attained education beyond 18 years or older, *n* = 1027), most commonly lived with a partner, spouse or civil partner (77%, *n* = 1624), most did not currently smoke (84.5%, *n* = 1789), and the majority were not on hormonal contraception (86.3%, *n* = 1821). The prevalence of irregular periods was fairly high (34.9%, *n* = 735), while endometriosis rates were below population prevalence (3.1%, *n* = 65) [64]. Mean BMI for the sample was 27.67 (SD = 5.88), indicating an average in the overweight range.

The average diet quality score was 6.37 (SD = 1.62), suggesting moderate adherence to a Mediterranean diet. Participants engaged in an average of 169.02 min (SD = 52.83) of total physical activity per day, including 76.95 min (SD = 27.91) of moderate-vigorous physical activity. The mean sleep duration was 7.65 h per night (SD = 1.09), sleep efficiency was 88.7% (SD = 8.29), and sleep regularity was 78.09% (SD = 12.34).

**Table 1:** Sample Characteristics of Included Participants (N=2109)

		N (%)	Mean (SD)
Irregular Periods	No	1374 (65.1%)	
	Yes	735 (34.9%)	
Endometriosis Diagnosis	No	2044 (96.9%)	
	Yes	65 (3.1%)	
Hormonal Contraception Use	No	1821 (86.3%)	
	Yes	288 (13.7%)	
Smoking Status	Every Day	229 (10.9%)	
	Occasionally	91 (4.3%)	
	Never	1112 (52.7%)	
	Used to	677 (32.1%)	
Highest Educational Qualification	Degree or above	673 (31.9%)	
	A Levels or equivalent	140 (6.6%)	
	Diploma	214 (10.1%)	
	GCSEs or equivalent	649 (30.8%)	
	No Academic Qualifications	433 (20.5%)	
Cohabiting Status	Living with civil partner	17 (0.8%)	
	Living with a partner	301 (14.3%)	
	Living with a spouse	1306 (61.9%)	
	Not living with a partner/no partner	485 (23%)	
Painful Periods	No	1178 (55.9%)	
	Yes	931 (44.1%)	
Heavy Periods	No	1016 (48.2%)	
	Yes	1093 (51.8%)	
PMS Symptoms	No	1065 (50.5%)	
	Yes	1044 (49.5%)	
Menstrual Symptom Occurrence	No menstrual symptoms	599 (28.4%)	
	One or two menstrual symptoms	975 (46.2%)	
	All three menstrual symptoms	535 (25.4%)	
Body Mass Index		27.67 (5.88)	N=2109
Mediterranean Diet Score		6.37 (1.62)	N=1929
Total Physical Activity (mins/day)		169.02 (52.83)	N=1722
Moderate-Vigorous Physical Activity (mins/day)		76.95 (27.91)	N=1722
Sleep Duration (hours)		7.65 (1.09)	N=1722
Sleep Efficiency (%)		88.7 (8.29)	N=1722
Sleep Regularity (%)		78.09 (12.34)	N=1722

Included sample = maximal sample, i.e. those with either complete diet diary data or complete activePAL4 data; General Wellbeing score derived from WEMWBS score [64]

### Associations between diet quality and menstrual symptoms

No significant associations were observed between diet quality and experiencing heavy or painful periods in either unadjusted or adjusted models (*p* > 0.05; Table S2). In the unadjusted model, participants in the lowest quartile of diet quality had reduced odds of experiencing PMS symptoms relative to those in the highest quartile (Odds ratio (OR): 0.67 (95% CI: 0.52,0.87, *p* = 0.002)), this association remained after adjusting for confounders (0.75(0.57, 0.98, *p* = 0.033)). A trend was observed in the same direction for those in the third quartile, though these associations were not statistically significant.

### Associations between device measured PA and menstrual symptoms

**Fig. 2:**
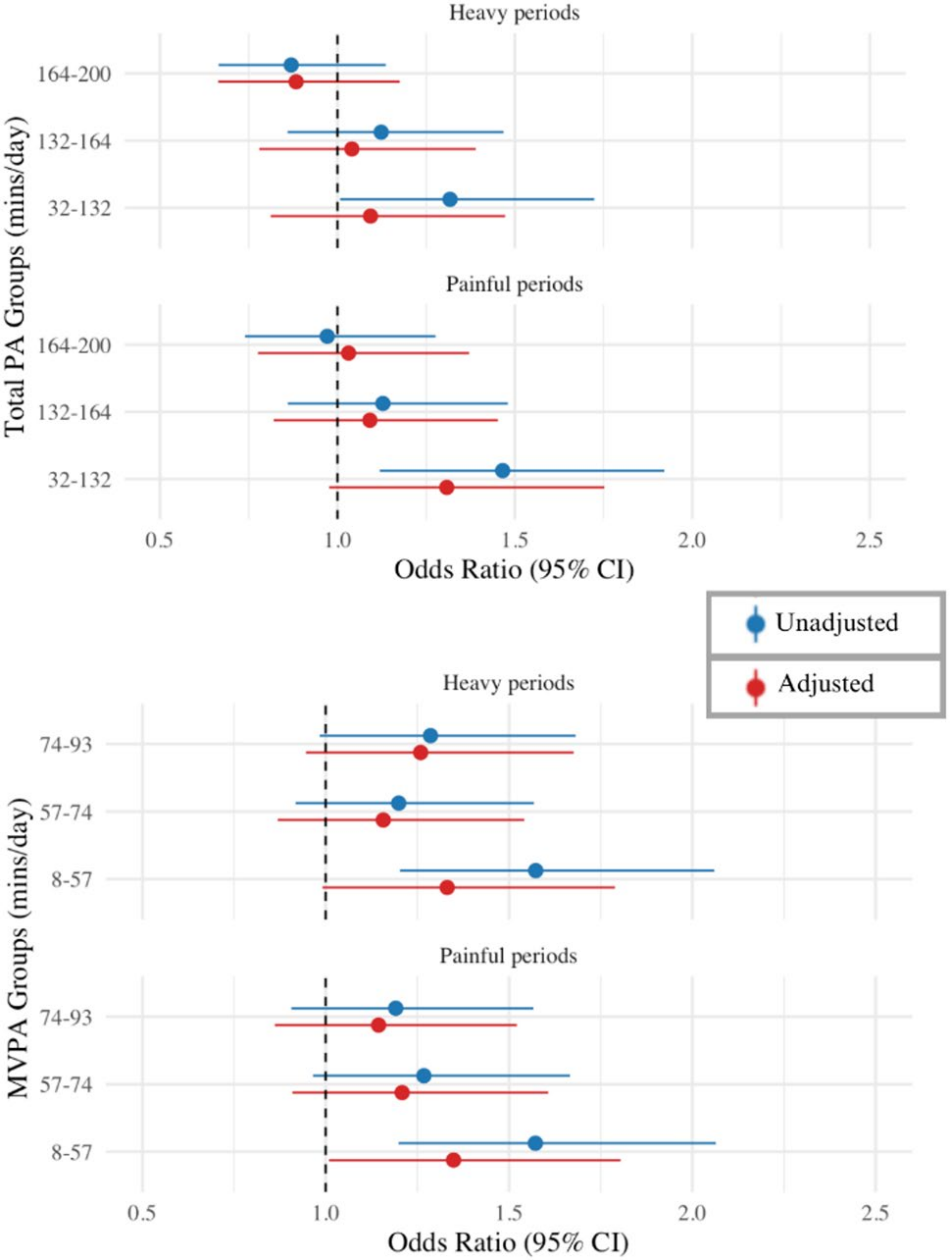
Odds of experiencing painful periods, heavy periods and PMS based on total physical activity and moderate-vigorous physical activity time/day in adjusted models (reference: healthiest group, total PA: 200–383 min/day; moderate-vigorous physical activity: 93–198 min/day, *N* = 1722, [OR: Odds ratio]

Lower levels of total physical activity were associated with increased odds of experiencing both painful and heavy periods. In unadjusted models, participants in the lowest quartile of total physical activity had 1.47 (1.12, 1.92) times higher odds of painful periods and 1.32 (1.01, 1.72) times higher odds of experiencing heavy periods compared to those in the highest quartile. These associations did not remain in adjusted models, though a trend towards significance was present for painful periods (1.31(0.98, 1.75) (Fig. 2). Lower moderate-vigorous physical activity levels were associated with increased risk of both painful and heavy periods. In unadjusted models, compared to the highest moderate-vigorous physical activity quartile, individuals in the lowest quartile had 1.57 (1.20, 2.06) times higher odds of having painful periods and 1.57 (1.20, 2.06) times higher odds of experiencing heavy periods. Estimates were somewhat attenuated in fully adjusted models, resulting in higher odds of 1.35 (1.01, 1.80) and 1.33 (0.99, 1.79), respectively. (Fig. 2). No significant associations were observed between total physical activity or moderate-vigorous physical activity and PMS symptoms (Table S2).

### Associations between device measured sleep and menstrual symptoms

No significant associations were observed between sleep duration quartiles and the odds of experiencing heavy periods, painful periods, or PMS symptoms (*p* > 0.05, Table S2). Similarly, sleep efficiency was not significantly associated with heavy periods or PMS symptoms (*p* > 0.05, Table S2). However, in the unadjusted model, participants in the lowest quartile of sleep efficiency had significantly increased odds of experiencing painful periods relative to those in the highest quartile (1.41(1.07, 1.84). This association was no longer significant in adjusted models (1.22 (0.91, 1.62)).

**Fig. 3:**
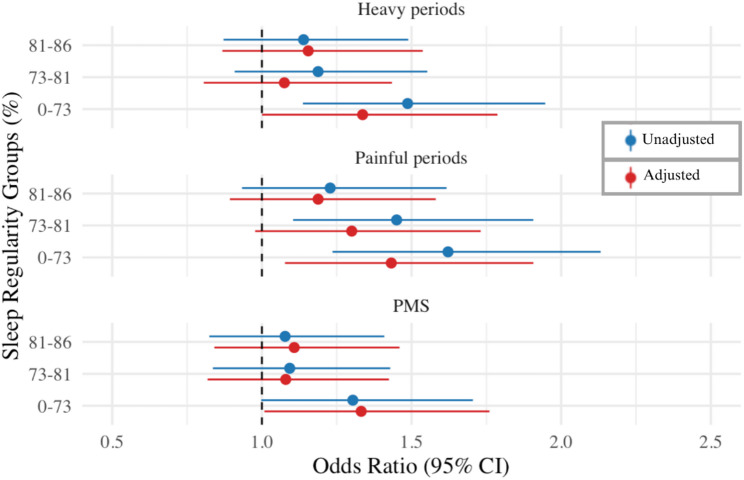
Odds of experiencing painful and heavy periods and PMS based on sleep regularity (reference: healthiest group i.e. 86–98% sleep regularity), *N* = 1722, [OR: Odds ratio]

Lower sleep regularity was associated with increased odds of experiencing all three menstrual symptoms in both unadjusted and adjusted models. In adjusted models, compared to participants in the highest quartile of sleep regularity, those in the lowest quartile had 1.43 (1.08, 1.91) times higher odds of experiencing painful periods, 1.34 (1.00, 1.79) times higher odds of experiencing heavy periods, and 1.33 (1.01, 1.76) times higher odds of experiencing PMS (Fig. 3, Table S2).

### Sensitivity analyses

Sensitivity analyses revealed significant differences across various demographic, lifestyle, and menstrual health-related variables between included and excluded participants (Table S3). Excluded participants were less likely to experience heavy periods (35.6% vs. 51.8%), painful periods (30.7% vs. 44.1%) and PMS 31.4% vs. 49.5%) symptoms. Included participants had better sleep efficiency (88.7% (SD = 8.29) vs. 87.32% (SD = 8.84)), sleep regularity (78.09% (SD = 12.34) vs. 76.72% (SD = 13.41)) and higher diet quality (6.37 (SD = 1.62) vs. 6.19 (SD = 1.68)) than excluded participants. There were no significant group differences found in total physical activity time, moderate-vigorous physical activity time, or sleep duration (Table S3). Finally, included participants were more likely to have never smoked (52.7% vs. 44.6%), had a lower prevalence of endometriosis diagnoses (3.1% vs. 5.7%), have a lower mean BMI (27.67 (SD = 5.88) vs. 28.94 (SD = 6.49)), and were less likely to not be living with a partner (23% vs. 29.7%).

The inclusion of number of children as a covariate resulted in similar associations to main models for diet and sleep variables, however associations with physical activity were further attenuated and no longer statistically significant in adjusted models (Table S4).

## Discussion

In a birth cohort study of women aged 46, lower levels of total physical activity and moderate-vigorous physical activity, as well as poorer sleep regularity were associated with greater odds of experiencing painful and heavy periods amongst women in midlife, with the most consistent dose-response relationships observed for painful periods. Unexpectedly, lower diet quality was associated with reduced odds of experiencing PMS symptoms in the present study. Overall, these findings highlight the potential impact of promoting physical activity and improving sleep regularity as an accessible, low-cost strategy to reduce the burden of menstrual symptoms, particularly during the perimenopausal life stage.

Although existing literature supports a positive role of adherence to a Mediterranean diet in reduced menstrual symptoms in younger women [20, 21] and postmenopausal women [24], findings amongst perimenopausal women have been mixed [65–67], highlighting potential challenges in accurately and meaningfully capturing diet quality during this life stage. Dietary behaviours are often variable and context-dependent, and the use of a single 24-hour dietary recall may not adequately reflect habitual intake, especially not across different stages of the menstrual cycle [68, 69], potentially influencing associations found presently. In addition, hormonal fluctuations characteristic of the perimenopausal transition may exert a stronger influence on menstrual symptom experience than diet quality alone, particularly when variability in dietary adherence is limited within the study population. Further research using repeated dietary assessments and longitudinal designs is needed to better understand how dietary behaviours interact with hormonal changes to influence menstrual experiences during the perimenopausal transition. This is a crucial lifestyle factor to explore further as it is well-established that the anti-inflammatory properties of Mediterranean-style dietary patterns can improve metabolic and cardiovascular health, and modulate oxidative stress and prostaglandin synthesis [70], all of which are important biological mechanisms involved in menstrual pain, bleeding, and premenstrual symptoms [71].

Meanwhile, findings linking physical activity and menstrual health symptoms are consistent with existing literature, with a recent meta-analysis highlighting a substantial increased risk of menstrual pain and PMS symptoms in those with lower physical activity frequency [25]. Notably, of 82 studies included in this review, only one utilised device-measured physical activity. As such, the present study addresses gaps in the literature by distinguishing between different physical activity intensities, revealing a potentially greater impact of moderate-vigorous physical activity compared to total physical activity on menstrual symptoms in midlife. The impact of sleep on menstrual health experiences is poorly understood; however, one recent systematic review of 35 studies, primarily reliant on self-reported sleep measures, did find consistent associations between participant’s sleep satisfaction, duration, efficiency, alertness during waking hours, and sleep latency and menstrual disturbance [31]. Similar to physical activity, the present study’s use of device-measured sleep metrics enables more accurate insights into the most impactful sleep characteristics on menstrual symptoms and suggests that sleep regularity contributes to menstrual health experiences, with no evidence for the role of sleep duration.

There are several biological mechanisms that may underlie the associations with physical activity observed in the present study. Moderate-vigorous physical activity is associated with improved hormonal regulation, lower levels of systemic inflammation and increased release of endorphins which can act as natural painkillers and mood stabilisers [72]. Thus, women who are inactive, or attaining exclusively light intensity physical activity such as gentle walking, may be less likely to experience the benefits of PA on their menstrual symptoms [73]. There is also some evidence to suggest that hormone cycles are intertwined with circadian rhythms which are responsible for regulating sleep [74, 75]. Disruption to normal sleep patterns, as demonstrated by studies of female shift workers, is associated with worsened menstrual burden [75], emphasising the importance of sleep regularity in managing menstrual symptoms. Furthermore, sleep disturbances can exacerbate central sensitisation and impair emotional regulation, which can alter symptom-perception, and intensify symptoms such as cramps, fatigue, and irritability [76]. Altogether, these pathways suggest plausible mechanisms by which modifiable lifestyle behaviours may influence menstrual symptoms during the perimenopausal transition.

### Implications and future directions

This study provides valuable insight into how lifestyle factors are linked to menstrual health in midlife. Lifestyle-based approaches to managing both menstrual and perimenopausal symptoms have risen exponentially, particularly due to the diverse challenges with seeking and accessing medical care [77]. The present study findings highlight the potential of lifestyle behavioural changes as accessible, non-pharmacological strategies to support menstrual health during the perimenopausal transition. Lifestyle interventions may also help reduce pressure on healthcare systems by enabling individuals to manage symptoms independently, rather than relying on long waiting times for medical care.

Future research should explore these associations over time using longitudinal designs and assess causality through intervention studies, such as randomised controlled trials of dietary, physical activity, or sleep-based strategies. Specifically, the present findings suggest that implementing interventions supporting more regular sleep patterns and providing more intensity-based physical activity protocols have potential to make the most impact. There is also a need to consider the interaction of common midlife experiences, such as increased care burden resulting from simultaneously dependent young children and elderly parents on women during this life stage, and how they may interact with debilitating symptom experiences related to the menopausal transition such as vasomotor symptoms and joint pain. Finally, it is also essential that future research addresses disparities by considering how socioeconomic and ethnicity factors shape both engagement with health behaviours and menstrual health outcomes, which was beyond the scope of the present study. Together, this future work has the potential to drastically improve the wellbeing of women in midlife.

### Strengths and limitations

PA and sleep data in the BCS70 dataset were captured using device-measured movement and derived using state-of-the-art algorithms, which is a key strength of this study compared to the reliance on self-reported physical activity and sleep in the menstrual health literature [25, 31]. The present study included a large sample relative to the small sample sizes characteristic of wider women’s health literature [78], allowing greater confidence in the generalisability of the findings. Moreover, the study has focused on the perimenopausal life stage which has been hugely under-researched. Finally, this study examined an age-homogenous sample, reducing age-related confounding which is crucial given the variability in menstrual symptoms across the reproductive lifespan [79].

There are also limitations to acknowledge. Notably, there were relatively high moderate-vigorous physical activity values observed in our sample, which may partly reflect the inclusion of fast walking, resulting in some higher-intensity low-intensity physical activity being classified as moderate-vigorous physical activity. Additionally, our accelerometer-based measurement captures short, incidental bursts of activity, such as climbing stairs or running for the bus, that are often missed in questionnaire-based assessments. Nonetheless, the levels of physical activity captured presently are consistent across five other studies that have processed the data using the same ActiPASS software [80]. Meanwhile, sample-bias analyses revealed potential selection bias, with excluded individuals displaying worse health behaviours and menstrual symptoms than included participants, likely biasing our results towards healthier individuals. Also, menstrual outcomes were measured using binary outcomes, which does not provide a comprehensive understanding of the severity or the extent of symptom burden on the individual’s quality of life or daily functioning. Furthermore, information on time since last menstrual period was not collected in this cohort, which may have introduced residual confounding, as cycle phase could influence lifestyle behaviours and symptom reporting. The poor quality of menstrual health measures, and women’s health data more generally, is a common limitation of population cohort datasets and highlights the need for more detailed and informative data on women’s health experiences within epidemiological studies [81, 82]. Similarly, PMS was measured via a single item, without distinguishing between the range of psychological and physical symptoms. Given different physiological pathways to diverse PMS symptoms, this may explain the unexpected lack of significant associations found in the present study in contrast to previous literature [83]. The results related to sleep should also be interpreted with caution, as accelerometer-derived variables are novel and thus are limited in which sleep components they can capture [84], as polysomnography remains the current gold-standard. Finally, the cross-sectional nature of the study does not allow conclusions to be made regarding causality, although, it is likely that there is a bidirectional relationship between lifestyle factors and menstrual health experiences.

## Conclusions

This study contributes to the growing body of evidence suggesting that healthy lifestyle behaviours could provide accessible and proactive approaches to menstrual symptom management during perimenopause. By incorporating consistent moderate-vigorous physical activity and promoting sleep regularity in daily routines, individuals may be able to reduce their symptom burden and improve overall wellbeing during this critical life stage. To facilitate the development of evidence-based solutions, further research is needed using rigorous methodology and incorporating co-production with lived experience individuals to ensure maximum benefits.

## Supplementary Information

Supplementary Material 1.

Supplementary Material 2.

## Data Availability

Original study protocol and survey documents can be found online at: https://bcs70.info and access to this data is available to bonified researchers through the UK Data Service repository [1970 British Cohort Study: 10.5255/UKDA-Series-200001].

## Abbreviations

PMS: Premenstrual syndrome
HRT: Hormone replacement therapy
