# Supplementary material for: Associations between physical activity, sleep patterns and diet quality and menstrual health symptoms in midlife: evidence from the 1970 British Cohort Study
Source: BMC Womens Health. 2026 May 26;26:375. doi: 10.1186/s12905-026-04533-9 (PMC13386675; doi:10.1186/s12905-026-04533-9)
Supplement: Supplementary file 1 — Supplementary Material 1. [file 12905_2026_4533_MOESM1_ESM.docx]

Supplemental Materials

Table S1 Mediterranean Diet Score Food Groups (Tao et al., 2025)

| **Food components** | **Food items** | **PyrMDS (0–15)** Serving required for the score of 0 | **PyrMDS (0–15)** Serving required for the score of 1 |
| --- | --- | --- | --- |
| **Vegetables** | raw salad, green leafy/cabbages, root vegetables, tomatoes, allium vegetables, other vegetables (including mushrooms), fruiting and mixed vegetables, vegetable side dishes, vegetable dips | 0/day | ≥6/day |
| **Legumes** | meat substitutes – soy, peas/sweetcorn, legumes & pulses | 0/week | ≥2/week |
| **Fruits** | citrus, berries, apples & pears, other fruit, dried fruit, stewed fruit | 0/day | 3–6/day |
| **Nuts** | salted nuts & seeds, unsalted nuts & seeds | 0/day | 1–2/day |
| **Cereals** | white bread, wholemeal bread, mixed (50/50), brown & seeded, other bread, bran cereal, biscuit cereal, oat cereal (non sugar), oat cereal (sugar), muesli, other cereal (sugar), white pasta & rice, wholemeal pasta, brown rice & other wholegrains, grain dishes – added fat | 0/day | 3–6/day |
| **Dairy** | whole milk, semi skimmed milk, skimmed milk, rice/oat milk, soy milk, full fat yogurt, low fat yogurt, high fat cheese, medium and low fat cheese | 0/day | 1.5–2.5/day |
| **Fish** | white fish & tinned tuna, shellfish, oily fish, breaded/battered fish | 0/week | ≥2/week |
| **Red meats** | pork, beef, lamb, other meat & offal | ≥4/week | <2/week |
| **Processed meats** | processed meat, breaded/battered chicken | ≥2/week | ≤1/week |
| **White meats** | poultry | 0/week | 1.5–2.5/week |
| **Eggs** | egg & egg dishes | 0/week | 2–4/week |
| **Potatoes** | potatoes/sweet potatoes (baked/boiled), mashed potatoes, fried/roast potatoes | ≥6/week | ≤3/week |
| **Wine** | white wine, red wine, fortified wine | ≥4/day for men,≥2/day for women | 1.5–2.5/day for men,0.5–1.5/day for women |
| **Sweets** | added sugars & preserves, chocolate confectionery, other sweets, biscuits, milk-dairy desserts, other desserts & cakes & pastries | ≥4/week | ≤2/week |
| **Fat** | olive oil | Non-consumers | Consumers |

A moderate consumption of fruits, nuts, cereals, dairy products, white meat, and eggs was recommended. Scoring was continuous and ranged from 0 (no intake) to 1 (meeting the recommended amount). If intake exceeded double the midpoint of the recommended range, it was considered excessive and scored a maximum of 0.5 points. Intakes between the recommended level and the overconsumption threshold were assigned points proportionally. Olive oil was an exception where participants are given either a discrete score of 0 or 1 depending on if they consumed any olive oil.

| **Table S2.** Associations between individual lifestyle behaviours & likelihood of menstrual health symptoms | | | | | | | | | | |
| --- | --- | --- | --- | --- | --- | --- | --- | --- | --- | --- |
|  |  | Painful Periods | | | Heavy Periods | | | PMS Symptoms | | |
|  | Model | OR | 95% CI | p-value | OR | 95% CI | p-value | OR | 95% CI | p-value |
| Mediterranean Diet | | | | | | | | | | |
| Q1 | Unadjusted | 1.04 | (0.81, 1.34) | 0.749 | 0.84 | (0.65, 1.07) | 0.162 | **0.70** | **(0.55, 0.90)** | **0.005** |
|  | Adjusted | 0.98 | (0.75, 1.29) | 0.912 | 0.83 | (0.63, 1.09) | 0.174 | 0.78 | (0.60, 1.02) | 0.070 |
| Q2 | Unadjusted | 1.04 | (0.81, 1.34) | 0.749 | 0.92 | (0.72, 1.18) | 0.525 | 0.84 | (0.66, 1.08) | 0.183 |
|  | Adjusted | 1.03 | (0.79, 1.34) | 0.817 | 0.93 | (0.71, 1.22) | 0.620 | 0.90 | (0.70, 1.17) | 0.440 |
| Q3 | Unadjusted | 0.97 | (0.75, 1.24) | 0.798 | 0.83 | (0.65, 1.07) | 0.144 | 0.82 | (0.64, 1.05) | 0.11 |
|  | Adjusted | 0.94 | (0.72, 1.22) | 0.651 | 0.80 | (0.62, 1.05) | 0.110 | 0.84 | (0.65, 1.08) | 0.18 |
| Total Physical Activity | | | | | | | | | | |
| Q1 | Unadjusted | **1.50** | **(1.15, 1.96)** | **0.003** | **1.32** | **(1.01, 1.72)** | **0.040** | 1.09 | (0.84, 1.42) | 0.52 |
|  | Adjusted | **1.33** | **(1.00, 1.77)** | **0.052** | 1.08 | (0.80, 1.44) | 0.62 | 1.14 | (0.86, 1.51) | 0.36 |
| Q2 | Unadjusted | 1.14 | (0.87, 1.49) | 0.34 | 1.10 | (0.85, 1.44) | 0.46 | 0.94 | (0.72, 1.22) | 0.64 |
|  | Adjusted | 1.10 | (0.83, 1.46) | 0.50 | 1.02 | (0.77, 1.35) | 0.91 | 0.93 | (0.71, 1.22) | 0.61 |
| Q3 | Unadjusted | 0.98 | (0.75, 1.28) | 0.89 | 0.86 | (0.66, 1.12) | 0.25 | 0.96 | (0.73, 1.24) | 0.74 |
|  | Adjusted | 1.02 | (0.77, 1.36) | 0.87 | 0.85 | (0.64, 1.13) | 0.26 | 1.00 | (0.76, 1.31) | 0.98 |
| MVPA | | | | | | | | | | |
| Q1 | Unadjusted | **1.67** | **(1.28, 2.18)** | **<0.001** | **1.61** | **(1.24, 2.10)** | **<0.001** | 1.10 | (0.84, 1.43) | 0.48 |
|  | Adjusted | **1.41** | **(1.06, 1.88)** | **0.018** | 1.32 | (0.99, 1.77) | 0.059 | 1.13 | (0.85, 1.49) | 0.41 |
| Q2 | Unadjusted | 1.29 | (0.99, 1.69) | 0.07 | 1.22 | (0.94, 1.59) | 0.14 | 1.05 | (0.80, 1.36) | 0.74 |
|  | Adjusted | 1.21 | (0.92, 1.61) | 0.18 | 1.16 | (0.87, 1.53) | 0.31 | 1.07 | (0.81, 1.40) | 0.63 |
| Q3 | Unadjusted | 1.23 | (0.94, 1.61) | 0.13 | **1.31** | **(1.01, 1.71)** | **0.044** | 1.02 | (0.78, 1.33) | 0.89 |
|  | Adjusted | 1.17 | (0.89, 1.55) | 0.27 | 1.27 | (0.96, 1.68) | 0.10 | 1.03 | (0.79, 1.35) | 0.82 |
| Sleep Duration | | | | | | | | | | |
| Q1 | Unadjusted | 0.99 | (0.76, 1.29) | 0.92 | 0.94 | (0.72, 1.23) | 0.66 | 0.90 | (0.69, 1.17) | 0.44 |
|  | Adjusted | 0.95 | (0.72, 1.25) | 0.71 | 0.91 | (0.69, 1.21) | 0.51 | 0.92 | (0.70, 1.20) | 0.54 |
| Q2 | Unadjusted | 0.93 | (0.71, 1.21) | 0.59 | 0.87 | (0.67, 1.14) | 0.31 | 1.18 | (0.90, 1.53) | 0.23 |
|  | Adjusted | 0.93 | (0.70, 1.22) | 0.60 | 0.87 | (0.66, 1.16) | 0.35 | 1.15 | (0.88, 1.51) | 0.31 |
| Q3 | Unadjusted | 0.85 | (0.65, 1.11) | 0.22 | 0.85 | (0.65, 1.11) | 0.23 | 1.10 | (0.85, 1.44) | 0.46 |
|  | Adjusted | 0.85 | (0.64, 1.12) | 0.25 | 0.86 | (0.65, 1.14) | 0.28 | 1.10 | (0.84, 1.44) | 0.49 |
| Sleep Efficiency | | | | | | | | | | |
| Q1 | Unadjusted | **1.42** | **(1.08, 1.85)** | **0.011** | 1.21 | (0.93, 1.58) | 0.149 | 1.00 | (0.76, 1.30) | 0.98 |
|  | Adjusted | 1.24 | (0.94, 1.65) | 0.13 | 1.04 | (0.78, 1.38) | 0.797 | 1.01 | (0.77, 1.32) | 0.96 |
| Q2 | Unadjusted | **1.40** | **(1.07, 1.83)** | **0.014** | 1.08 | (0.83, 1.41) | 0.545 | 1.08 | (0.83, 1.40) | 0.59 |
|  | Adjusted | 1.24 | (0.94, 1.65) | 0.13 | 0.92 | (0.69, 1.22) | 0.55 | 1.05 | (0.80, 1.38) | 0.71 |
| Q3 | Unadjusted | 1.20 | (0.91, 1.57) | 0.19 | 1.09 | (0.84, 1.42) | 0.50 | 1.13 | (0.87, 1.48) | 0.35 |
|  | Adjusted | 1.19 | (0.90, 1.57) | 0.23 | 1.05 | (0.79, 1.39) | 0.73 | 1.14 | (0.87, 1.49) | 0.35 |
| Sleep Regularity | | | | | | | | | | |
| Q1 | Unadjusted | **1.69** | **(1.29, 2.21)** | **<0.001** | **1.52** | **(1.17, 1.99)** | **0.002** | **1.33** | **(1.02, 1.73)** | **0.034** |
|  | Adjusted | **1.48** | **(1.12, 1.96)** | **0.006** | **1.36** | **(1.02, 1.81)** | **0.036** | **1.36** | **(1.03, 1.79)** | **0.029** |
| Q2 | Unadjusted | **1.50** | **(1.14, 1.96)** | **0.003** | 1.23 | (0.95, 1.60) | 0.12 | 1.09 | (0.84, 1.43) | 0.50 |
|  | Adjusted | **1.34** | **(1.01, 1.78)** | **0.041** | 1.11 | (0.84, 1.48) | 0.47 | 1.08 | (0.82, 1.42) | 0.59 |
| Q3 | Unadjusted | 1.27 | (0.97, 1.66) | 0.09 | 1.19 | (0.91, 1.55) | 0.20 | 1.11 | (0.86, 1.45) | 0.42 |
|  | Adjusted | 1.22 | (0.92, 1.61) | 0.17 | 1.20 | (0.90, 1.59) | 0.21 | 1.14 | (0.87, 1.50) | 0.33 |

| **Table S3.** Sensitivity Analysis of Sample Characteristics (Excluded vs Included) | | | | |
| --- | --- | --- | --- | --- |
|  | | Included Participants  (N = 2176) | Excluded Sample  (N = 2250) |  |
| **Categorical Variables** | | | | |
|  |  | N (%) | N (%) | p-value |
| Irregular Periods | No | 1403 (64.5%) | 1510 (67.1%) | 0.002** |
|  | Yes | 773 (35.5%) | 680 (30.2%) |  |
| Endometriosis Diagnosis | No | 2106 (96.8%) | 2043 (90.8%) | <0.001*** |
|  | Yes | 69 (3.2%) | 128 (5.7%) |  |
| Hormonal Contraception Use | No | 1882 (86.5%) | 1530 (68%) | <0.001*** |
|  | Yes | 294 (13.5%) | 525 (23.3%) |  |
| Smoking Status | Never | 1137 (52.3%) | 1009 (44.8%) | <0.001*** |
|  | Used to | 707 (32.5%) | 693 (30.8%) |  |
|  | Occasionally | 94 (4.3%) | 120 (5.3%) |  |
|  | Every Day | 238 (10.9%) | 427 (19%) |  |
| Highest Educational Qualification | No Academic Qualifications | 448 (20.6%) | 679 (30.2%) | <0.001*** |
|  | GCSEs or equivalent | 676 (31.1%) | 732 (32.5%) |  |
|  | A Levels or equivalent | 143 (6.6%) | 105 (4.7%) |  |
|  | Diploma | 222 (10.2%) | 203 (9%) |  |
|  | Degree or above | 687 (31.6%) | 467 (20.8%) |  |
| Heavy Periods | No | 1043 (47.9%) | 1413 (62.8%) | <0.001*** |
|  | Yes | 1133 (52.1%) | 784 (34.8%) |  |
| Painful Periods | No | 1212 (55.7%) | 1515 (67.3%) | <0.001*** |
|  | Yes | 964 (44.3%) | 679 (30.2%) |  |
| PMS Symptoms | No | 1092 (50.2%) | 1498 (66.6%) | <0.001*** |
|  | Yes | 1084 (49.8%) | 687 (30.5%) |  |
| **Continuous Variables** | | | |  |
|  | | Mean (SD), N included | Mean (SD), N included | p-value |
| General Wellbeing | | 71.63 (20.86), N=2141 | 63.18 (24.64), N=2045 | <0.001*** |
| Body Mass Index | | 27.68 (5.95), N=2176 | 28.97 (6.42), N=1649 | <0.001*** |
| Diet Quality | | 6.39 (1.62), N=1988 | 6.16 (1.67), N=1166 | <0.001*** |
| Total Physical Activity (mins/day) | | 169 (52.97), N=1773 | 166.85 (58.14), N=1031 | 0.330 |
| Moderate-Vigorous Physical Activity (mins/day) | | 76.85 (27.99), N=1773 | 75.6 (29.74), N=1031 | 0.270 |
| Sleep Duration (hours) | | 7.66 (1.09), N=1773 | 7.58 (1.21), N=1031 | 0.085 |
| Sleep Efficiency (%) | | 88.7 (8.29), N=1773 | 87.25 (8.87), N=1031 | <0.001*** |
| Sleep Regularity (%) | | 78.03 (12.46), N=1773 | 76.75 (13.27), N=1031 | 0.012 * |
| *Included sample = maximal sample, i.e. people who had either complete diet diary data or complete activepal4 data*  *Excluded participants have some missing data throughout*  ** p <0.05, **p<0.01, ***p<0.001* | | | | |
