# Supplementary material for: Associations between physical activity, sleep patterns and diet quality and menstrual health symptoms in midlife: evidence from the 1970 British Cohort Study
Source: BMC Womens Health. 2026 May 26;26:375. doi: 10.1186/s12905-026-04533-9 (PMC13386675; doi:10.1186/s12905-026-04533-9)
Supplement: Supplementary file 2 — Supplementary Material 2. [file 12905_2026_4533_MOESM2_ESM.docx]

Supplemental Materials

Table S1 Mediterranean Diet Score Food Groups (Tao et al., 2025)

| **Food components** | **Food items** | **PyrMDS (0–15)** Serving required for the score of 0 | **PyrMDS (0–15)** Serving required for the score of 1 |
| --- | --- | --- | --- |
| **Vegetables** | raw salad, green leafy/cabbages, root vegetables, tomatoes, allium vegetables, other vegetables (including mushrooms), fruiting and mixed vegetables, vegetable side dishes, vegetable dips | 0/day | ≥6/day |
| **Legumes** | meat substitutes – soy, peas/sweetcorn, legumes & pulses | 0/week | ≥2/week |
| **Fruits** | citrus, berries, apples & pears, other fruit, dried fruit, stewed fruit | 0/day | 3–6/day |
| **Nuts** | salted nuts & seeds, unsalted nuts & seeds | 0/day | 1–2/day |
| **Cereals** | white bread, wholemeal bread, mixed (50/50), brown & seeded, other bread, bran cereal, biscuit cereal, oat cereal (non sugar), oat cereal (sugar), muesli, other cereal (sugar), white pasta & rice, wholemeal pasta, brown rice & other wholegrains, grain dishes – added fat | 0/day | 3–6/day |
| **Dairy** | whole milk, semi skimmed milk, skimmed milk, rice/oat milk, soy milk, full fat yogurt, low fat yogurt, high fat cheese, medium and low fat cheese | 0/day | 1.5–2.5/day |
| **Fish** | white fish & tinned tuna, shellfish, oily fish, breaded/battered fish | 0/week | ≥2/week |
| **Red meats** | pork, beef, lamb, other meat & offal | ≥4/week | <2/week |
| **Processed meats** | processed meat, breaded/battered chicken | ≥2/week | ≤1/week |
| **White meats** | poultry | 0/week | 1.5–2.5/week |
| **Eggs** | egg & egg dishes | 0/week | 2–4/week |
| **Potatoes** | potatoes/sweet potatoes (baked/boiled), mashed potatoes, fried/roast potatoes | ≥6/week | ≤3/week |
| **Wine** | white wine, red wine, fortified wine | ≥4/day for men,≥2/day for women | 1.5–2.5/day for men,0.5–1.5/day for women |
| **Sweets** | added sugars & preserves, chocolate confectionery, other sweets, biscuits, milk-dairy desserts, other desserts & cakes & pastries | ≥4/week | ≤2/week |
| **Fat** | olive oil | Non-consumers | Consumers |

A moderate consumption of fruits, nuts, cereals, dairy products, white meat, and eggs was recommended. Scoring was continuous and ranged from 0 (no intake) to 1 (meeting the recommended amount). If intake exceeded double the midpoint of the recommended range, it was considered excessive and scored a maximum of 0.5 points. Intakes between the recommended level and the overconsumption threshold were assigned points proportionally. Olive oil was an exception where participants are given either a discrete score of 0 or 1 depending on if they consumed any olive oil.

| **Table S2.** Associations between individual lifestyle behaviours & likelihood of menstrual health symptoms | | | | | | | | | |  |
| --- | --- | --- | --- | --- | --- | --- | --- | --- | --- | --- |
|  |  | Painful Periods | | | Heavy Periods | | | PMS Symptoms | | |
|  | Model | OR | 95% CI | p-value | OR | 95% CI | p-value | OR | 95% CI | p-value |
| Mediterranean Diet (N=1929) | | | | | | | | | |  |
| Q1 | Unadjusted | 1.03 | (0.80, 1.33) | 0.83 | 0.82 | (0.64, 1.06) | 0.13 | **0.67** | **(0.52, 0.87)** | **<0.01** |
| Q1 | Adjusted | 0.97 | (0.74, 1.27) | 0.81 | 0.80 | (0.61, 1.05) | 0.11 | **0.75** | **(0.57, 0.98)** | **0.03** |
| Q2 | Unadjusted | 1.05 | (0.82, 1.36) | 0.70 | 0.94 | (0.73, 1.21) | 0.61 | 0.85 | (0.66, 1.09) | 0.20 |
| Q2 | Adjusted | 1.05 | (0.80, 1.37) | 0.75 | 0.95 | (0.72, 1.25) | 0.70 | 0.90 | (0.69, 1.17) | 0.42 |
| Q3 | Unadjusted | 0.97 | (0.75, 1.25) | 0.80 | 0.83 | (0.65, 1.07) | 0.16 | 0.79 | (0.61, 1.01) | 0.06 |
| Q3 | Adjusted | 0.93 | (0.71, 1.22) | 0.61 | 0.80 | (0.61, 1.05) | 0.11 | 0.80 | (0.61, 1.03) | 0.09 |
| Total Physical Activity (N=1722) | | | | | | | | | |  |
| Q1 | Unadjusted | **1.47** | **(1.12, 1.92)** | **<0.01** | **1.32** | **(1.01, 1.72)** | **0.04** | 1.04 | (0.80, 1.36) | 0.76 |
| Q1 | Adjusted | 1.31 | (0.98, 1.75) | 0.07 | 1.09 | (0.81, 1.47) | 0.56 | 1.09 | (0.82, 1.45) | 0.56 |
| Q2 | Unadjusted | 1.13 | (0.86, 1.48) | 0.38 | 1.12 | (0.86, 1.47) | 0.40 | 0.92 | (0.71, 1.21) | 0.56 |
| Q2 | Adjusted | 1.09 | (0.82, 1.45) | 0.55 | 1.04 | (0.78, 1.39) | 0.79 | 0.91 | (0.69, 1.20) | 0.50 |
| Q3 | Unadjusted | 0.97 | (0.74, 1.28) | 0.83 | 0.87 | (0.67, 1.14) | 0.31 | 0.95 | (0.72, 1.24) | 0.68 |
| Q3 | Adjusted | 1.03 | (0.78, 1.37) | 0.83 | 0.88 | (0.66, 1.18) | 0.40 | 1.00 | (0.76, 1.31) | 0.98 |
| Moderate-vigorous physical activity (N=1722) | | | | | | | | | |  |
| Q1 | Unadjusted | **1.57** | **(1.20, 2.06)** | **<0.001** | **1.57** | **(1.20, 2.06)** | **<0.001** | 1.03 | (0.79, 1.35) | 0.81 |
| Q1 | Adjusted | **1.35** | **(1.01, 1.80)** | **0.04** | 1.33 | (0.99, 1.79) | 0.06 | 1.06 | (0.80, 1.40) | 0.70 |
| Q2 | Unadjusted | 1.27 | (0.97, 1.67) | 0.09 | 1.20 | (0.92, 1.57) | 0.18 | 1.00 | (0.76, 1.30) | 0.97 |
| Q2 | Adjusted | 1.21 | (0.91, 1.61) | 0.19 | 1.16 | (0.87, 1.54) | 0.32 | 1.02 | (0.77, 1.34) | 0.91 |
| Q3 | Unadjusted | 1.19 | (0.91, 1.57) | 0.21 | 1.29 | (0.98, 1.68) | 0.07 | 0.99 | (0.76, 1.29) | 0.95 |
| Q3 | Adjusted | 1.14 | (0.86, 1.52) | 0.35 | 1.26 | (0.95, 1.68) | 0.11 | 1.01 | (0.77, 1.33) | 0.95 |
| Sleep Duration (N=1722) | | | | | | | | | |  |
| Q1 | Unadjusted | 1.01 | (0.77, 1.32) | 0.97 | 0.94 | (0.72, 1.23) | 0.66 | 0.88 | (0.67, 1.15) | 0.36 |
| Q1 | Adjusted | 0.97 | (0.73, 1.28) | 0.81 | 0.90 | (0.68, 1.20) | 0.49 | 0.90 | (0.68, 1.19) | 0.47 |
| Q2 | Unadjusted | 0.95 | (0.73, 1.24) | 0.71 | 0.88 | (0.67, 1.15) | 0.36 | 1.22 | (0.93, 1.60) | 0.14 |
| Q2 | Adjusted | 0.95 | (0.71, 1.25) | 0.70 | 0.87 | (0.66, 1.16) | 0.36 | 1.21 | (0.92, 1.59) | 0.18 |
| Q3 | Unadjusted | 0.85 | (0.65, 1.11) | 0.24 | 0.85 | (0.65, 1.12) | 0.25 | 1.06 | (0.81, 1.38) | 0.68 |
| Q3 | Adjusted | 0.85 | (0.64, 1.12) | 0.24 | 0.85 | (0.64, 1.13) | 0.26 | 1.05 | (0.80, 1.39) | 0.71 |
| Sleep Efficiency (N=1722) | | | | | | | | | |  |
| Q1 | Unadjusted | **1.41** | **(1.07, 1.84)** | **0.01** | 1.21 | (0.93, 1.58) | 0.16 | 1.00 | (0.76, 1.30) | 0.97 |
| Q1 | Adjusted | 1.22 | (0.91, 1.62) | 0.18 | 1.02 | (0.76, 1.37) | 0.88 | 1.00 | (0.75, 1.32) | 0.98 |
| Q2 | Unadjusted | 1.35 | (1.03, 1.78) | 0.03 | 1.03 | (0.79, 1.35) | 0.81 | 1.06 | (0.81, 1.39) | 0.66 |
| Q2 | Adjusted | 1.20 | (0.90, 1.59) | 0.22 | 0.86 | (0.64, 1.14) | 0.30 | 1.04 | (0.79, 1.37) | 0.78 |
| Q3 | Unadjusted | 1.20 | (0.92, 1.58) | 0.19 | 1.07 | (0.82, 1.39) | 0.63 | 1.16 | (0.89, 1.52) | 0.28 |
| Q3 | Adjusted | 1.17 | (0.89, 1.56) | 0.265 | 1.00 | (0.76, 1.33) | 0.974 | 1.16 | (0.88, 1.53) | 0.291 |
| Sleep Regularity (N=1722) | | | | | | | | | |  |
| Q1 | Unadjusted | **1.62** | **(1.24, 2.13)** | **<0.001** | **1.49** | **(1.14, 1.95)** | **<0.01** | **1.30** | **(1.00, 1.71)** | **0.05** |
| Q1 | Adjusted | **1.43** | **(1.08, 1.91)** | **0.01** | **1.34** | **(1.00, 1.79)** | **0.05** | **1.33** | **(1.01, 1.76)** | **0.04** |
| Q2 | Unadjusted | **1.45** | **(1.10, 1.91)** | **<0.01** | 1.19 | (0.91, 1.55) | 0.21 | 1.09 | (0.84, 1.43) | 0.52 |
| Q2 | Adjusted | 1.30 | (0.98, 1.73) | 0.07 | 1.08 | (0.81, 1.43) | 0.62 | 1.08 | (0.82, 1.42) | 0.59 |
| Q3 | Unadjusted | 1.23 | (0.93, 1.62) | 0.14 | 1.14 | (0.87, 1.49) | 0.34 | 1.08 | (0.82, 1.41) | 0.59 |
| Q3 | Adjusted | 1.19 | (0.89, 1.58) | 0.24 | 1.15 | (0.87, 1.54) | 0.32 | 1.11 | (0.84, 1.46) | 0.46 |
| *N.B. Reference category for all models is Q4, representing the healthiest group (i.e. highest diet quality, most physical activity time, highest sleep quality)* | | | | | | | | | | |

| **Table S3.** Sensitivity Analysis of Sample Characteristics (Excluded vs Included) | | | | | |
| --- | --- | --- | --- | --- | --- |
|  |  | Included Participants (N = 2109) | Excluded Sample  (N = 2317) |  |  |
| **Categorical Variables** | | | | | |
|  |  | N (%) | N (%) | p-value |  |
| Irregular Periods | No | 1374 (65.1%) | 1539 (66.4%) | 0.04 * |  |
|  | Yes | 735 (34.9%) | 718 (31%) | 0.04 * |  |
| Endometriosis Diagnosis | No | 2044 (96.9%) | 2105 (90.9%) | <0.001*** |  |
|  | Yes | 65 (3.1%) | 132 (5.7%) | <0.001*** |  |
| Hormonal Contraception Use | No | 1821 (86.3%) | 1591 (68.7%) | <0.001*** |  |
|  | Yes | 288 (13.7%) | 531 (22.9%) | <0.001*** |  |
| Smoking Status | Every Day | 229 (10.9%) | 436 (18.8%) | <0.001*** |  |
|  | Never | 1112 (52.7%) | 1034 (44.6%) | <0.001*** |  |
|  | Occasionally | 91 (4.3%) | 123 (5.3%) | <0.001*** |  |
|  | Used to | 677 (32.1%) | 723 (31.2%) | <0.001*** |  |
| Highest Educational Qualification | A Levels or equivalent | 140 (6.6%) | 108 (4.7%) | <0.001*** |  |
|  | Degree or above | 673 (31.9%) | 481 (20.8%) | <0.001*** |  |
|  | Diploma | 214 (10.1%) | 211 (9.1%) | <0.001*** |  |
|  | GCSEs or equivalent | 649 (30.8%) | 759 (32.8%) | <0.001*** |  |
|  | No Academic Qualifications | 433 (20.5%) | 694 (30%) | <0.001*** |  |
| Cohabiting Status | Living with civil partner | 17 (0.8%) | 9 (0.4%) | <0.001*** |  |
|  | Living with a partner | 301 (14.3%) | 324 (14%) | <0.001*** |  |
|  | Living with a spouse | 1306 (61.9%) | 1296 (55.9%) | <0.001*** |  |
|  | Not living with a partner/no partner | 485 (23%) | 688 (29.7%) | <0.001*** |  |
| Heavy Periods | No | 1016 (48.2%) | 1440 (62.1%) | <0.001*** |  |
|  | Yes | 1093 (51.8%) | 824 (35.6%) | <0.001*** |  |
| Painful Periods | No | 1178 (55.9%) | 1549 (66.9%) | <0.001*** |  |
|  | Yes | 931 (44.1%) | 712 (30.7%) | <0.001*** |  |
| PMS Symptoms | No | 1065 (50.5%) | 1525 (65.8%) | <0.001*** |  |
|  | Yes | 1044 (49.5%) | 727 (31.4%) | <0.001*** |  |
| **Continuous Variables** | | | | | |
| General Wellbeing |  | 71.81 (20.72), N=2076 | 63.26 (24.64), N=2110 | <0.001*** |  |
| Body Mass Index |  | 27.67 (5.88), N=2109 | 28.94 (6.49), N=1716 | <0.001*** |  |
| Mediterranean Diet Score |  | 6.37 (1.62), N=1929 | 6.19 (1.68), N=1225 | 0.003 ** |  |
| Total Physical Activity (mins/day) |  | 169.02 (52.83), N=1722 | 166.92 (58.11), N=1082 | 0.34 |  |
| Moderate-Vigorous Physical Activity (mins/day) |  | 76.95 (27.91), N=1722 | 75.5 (29.77), N=1082 | 0.20 |  |
| Sleep Duration (hours) |  | 7.65 (1.09), N=1722 | 7.59 (1.2), N=1082 | 0.19 |  |
| Sleep Efficiency (%) |  | 88.7 (8.29), N=1722 | 87.32 (8.84), N=1082 | <0.001*** |  |
| Sleep Regularity (%) |  | 78.09 (12.34), N=1722 | 76.72 (13.41), N=1082 | <0.01 ** |  |
| *Included sample = maximal sample, i.e. people who had either complete diet diary data or complete activepal4 data*  *Excluded participants have some missing data throughout*  ** p <0.05, **p<0.01, ***p<0.001* | | | | | |

| **Table S4**. Main models additionally adjusted for number of children | | | | | | | | | | | | | | | | | | | | | | | | |
| --- | --- | --- | --- | --- | --- | --- | --- | --- | --- | --- | --- | --- | --- | --- | --- | --- | --- | --- | --- | --- | --- | --- | --- | --- |
|  |  | Painful Periods | | | | | | | Heavy Periods | | | | | | | PMS Symptoms | | | | | | | | |
|  | Model | OR | | 95% CI | | p-value | | OR | | | 95% CI | | p-value | | OR | | | 95% CI | | | p-value | | |  |
| Mediterranean Diet (N=1929) | | | | | | | | | | | | | | | | | | | | | | | | |
| Q1 | Adjusted | 0.98 | | (0.74, 1.28) | | 0.87 | | 0.80 | | | (0.60, 1.05) | | 0.11 | | **0.75** | | | **(0.57, 0.98)** | **0.033** | | |  |  |  |
| Q2 | Adjusted | 1.04 | | (0.80, 1.36) | | 0.76 | | 0.95 | | | (0.72, 1.25) | | 0.71 | | 0.90 | | | (0.69, 1.17) | 0.43 | | |  |  |  |
| Q3 | Adjusted | 0.94 | | (0.72, 1.22) | | 0.63 | | 0.80 | | | (0.61, 1.05) | | 0.11 | | 0.80 | | | (0.61, 1.03) | 0.09 | | |  |  |  |
| Total Physical Activity (N=1722) | | | | | | | | | | | | | | | | | | | | | | | | |
| Q1 | Adjusted | 1.18 | (0.88, 1.60) | | 0.27 | | 1.09 | | | (0.81, 1.48) | | 0.57 | | 1.08 | | | (0.81, 1.45) | | | 0.59 | | |  |  |
| Q2 | Adjusted | 1.05 | (0.79, 1.40) | | 0.74 | | 1.04 | | | (0.78, 1.39) | | 0.79 | | 0.91 | | | (0.69, 1.20) | | | 0.49 | | |  |  |
| Q3 | Adjusted | 0.99 | (0.75, 1.32) | | 0.96 | | 0.88 | | | (0.66, 1.18) | | 0.39 | | 0.99 | | | (0.75, 1.31) | | | 0.97 | | |  |  |
| MVPA (N=1722) | | | | | | | | | | | | | | | | | | | | | | | | |
| Q1 | Adjusted | 1.29 | | (0.96, 1.73) | | 0.09 | | 1.33 | | | (0.99, 1.79) | | 0.06 | | 1.05 | | | (0.79, 1.40) | | | 0.72 | | |  |
| Q2 | Adjusted | 1.19 | | (0.90, 1.59) | | 0.23 | | 1.16 | | | (0.87, 1.54) | | 0.32 | | 1.01 | | | (0.77, 1.34) | | | 0.92 | | |  |
| Q3 | Adjusted | 1.16 | | (0.87, 1.55) | | 0.30 | | 1.26 | | | (0.95, 1.68) | | 0.11 | | 1.01 | | | (0.77, 1.33) | | | 0.94 | | |  |
| Sleep Duration (N=1722) | | | | | | | | | | | | | | | | | | | | | | | | |
| Q1 | Adjusted | 1.02 | | (0.77, 1.36) | | 0.87 | | 0.90 | | | (0.68, 1.21) | | 0.50 | | 0.91 | | | (0.69, 1.20) | | | 0.49 | | |  |
| Q2 | Adjusted | 0.99 | | (0.75, 1.32) | | 0.95 | | 0.88 | | | (0.66, 1.17) | | 0.37 | | 1.21 | | | (0.92, 1.60) | | | 0.18 | | |  |
| Q3 | Adjusted | 0.89 | | (0.67, 1.18) | | 0.42 | | 0.85 | | | (0.64, 1.13) | | 0.27 | | 1.06 | | | (0.80, 1.39) | | | 0.69 | | |  |
| Sleep Efficiency (N=1722) | | | | | | | | | | | | | | | | | | | | | | | | |
| Q1 | Adjusted | 1.16 | | (0.87, 1.55) | | 0.32 | | 1.02 | | | (0.76, 1.36) | | 0.90 | | 0.99 | | | (0.75, 1.32) | | | 0.96 | | |  |
| Q2 | Adjusted | 1.15 | | (0.87, 1.54) | | 0.32 | | 0.86 | | | (0.64, 1.14) | | 0.29 | | 1.04 | | | (0.79, 1.37) | | | 0.80 | | |  |
| Q3 | Adjusted | 1.14 | | (0.86, 1.51) | | 0.376 | | 1.00 | | | (0.75, 1.33) | | 0.987 | | 1.16 | | | (0.88, 1.52) | | | 0.300 | | |  |
| Sleep Regularity (N=1722) | | | | | | | | | | | | | | | | | | | | | | | | |
| Q1 | Adjusted | **1.37** | | **(1.03, 1.82)** | | **0.03** | | 1.34 | | | (1.00, 1.79) | | 0.05 | | **1.33** | | | **(1.01, 1.76)** | | | **0.04** | | |  |
| Q2 | Adjusted | 1.30 | | (0.98, 1.73) | | 0.07 | | 1.08 | | | (0.81, 1.43) | | 0.62 | | 1.08 | | | (0.82, 1.42) | | | 0.59 | | |  |
| Q3 | Adjusted | 1.20 | | (0.90, 1.60) | | 0.21 | | 1.15 | | | (0.87, 1.54) | | 0.32 | | 1.11 | | | (0.84, 1.46) | | | 0.47 | | |  |
| *N.B.*  *(1) Reference category for all models is Q4, representing the healthiest group (i.e. highest diet quality, most physical activity time, highest sleep quality)*  *(2) Sample size did not change following the inclusion of number of children as a covariate thus only adjusted models are presented above.* | | | | | | | | | | | | | | | | | | | | | | | |  |
